# Predicting Postoperative Hospital Stays Using Nursing Narratives and the Reverse Time Attention (RETAIN) Model: Retrospective Cohort Study

**DOI:** 10.2196/45377

**Published:** 2023-12-19

**Authors:** Sungjoo Han, Yong Bum Kim, Jae Hong No, Dong Hoon Suh, Kidong Kim, Soyeon Ahn

**Affiliations:** 1 Division of Statistics, Medical Research Collaborating Center, Seoul National University Bundang Hospital, Seongnam, Republic of Korea; 2 Department of Obstetrics and Gynecology, Seoul National University Bundang Hospital, Seongnam, Republic of Korea

**Keywords:** discharge prediction, text mining, free text, extraction, length of stay, hospital stay, electronic health record, EHR, discharge, interpretable deep learning, risk prediction, nursing, machine learning, deep learning, predict, ovarian cancer

## Abstract

**Background:**

Nursing narratives are an intriguing feature in the prediction of short-term clinical outcomes. However, it is unclear which nursing narratives significantly impact the prediction of postoperative length of stay (LOS) in deep learning models.

**Objective:**

Therefore, we applied the Reverse Time Attention (RETAIN) model to predict LOS, entering nursing narratives as the main input.

**Methods:**

A total of 354 patients who underwent ovarian cancer surgery at the Seoul National University Bundang Hospital from 2014 to 2020 were retrospectively enrolled. Nursing narratives collected within 3 postoperative days were used to predict prolonged LOS (≥10 days). The physician’s assessment was conducted based on a retrospective review of the physician’s note within the same period of the data model used.

**Results:**

The model performed better than the physician’s assessment (area under the receiver operating curve of 0.81 vs 0.58; *P*=.02). Nursing narratives entered on the first day were the most influential predictors in prolonged LOS. The likelihood of prolonged LOS increased if the physician had to check the patient often and if the patient received intravenous fluids or intravenous patient-controlled analgesia late.

**Conclusions:**

The use of the RETAIN model on nursing narratives predicted postoperative LOS effectively for patients who underwent ovarian cancer surgery. These findings suggest that accurate and interpretable deep learning information obtained shortly after surgery may accurately predict prolonged LOS.

## Introduction

Postoperative length of stay (LOS) is an important indicator of hospital management efficiency. A precise estimate of LOS optimizes hospital bed availability and resource allocation, thereby improving health outcomes and lowering costs [1,2]. There is an increasing need to predict LOS using electronic health records (EHRs) with machine learning methods [3-6]. EHRs contain data on patients’ demographics, diagnoses, medications, vital signs, and laboratory results, which are fed into deep learning algorithms. For example, Safavi et al [7] have suggested a feedforward neural network model comprising clinical and administrative data extracted from EHRs to predict discharge from inpatient surgical care. Zhang et al [8] have investigated a prediction model for next-day discharge using EHR access logs combined with gradient-boosted ensembles of decision trees. For this study, we refer to Stone et al [9] for a comprehensive review of the prediction of hospital LOS.

We focused on nursing narratives in EHRs as a promising predictor of postoperative LOS. Nursing narratives are representations of the nursing process and contain data regarding when and how nursing actions are performed on patients [10,11]. Analyses of nursing notes using machine learning models have shown promising results in predicting short-term patient outcomes [12,13]. We have previously reported that a deep learning model based on nursing narratives can effectively predict postoperative LOS [14]. However, a fundamental problem of deep learning models is their lack of interpretability, which restrains their clinical applicability [15,16]. Moreover, our previous study implemented long short-term memory using frequencies of individual nursing narrative entries for 5 postoperative days as features, which limited the power of dependencies between time steps of sequence data.

To overcome this issue, various interpretable artificial intelligence models have been examined [17]. The Reverse Time Attention (RETAIN) model is an interpretable predictive model developed for application with EHR data. RETAIN’s major advantage is its high accuracy while remaining clinically interpretable by adapting a 2-level neural attention mechanism in a recurrent neural network architecture. Consequently, RETAIN can detect both influential nurse visits and clinical features [16]. Several studies have demonstrated the clinical utility of the RETAIN model in diverse clinical contexts [18-23]. AlSaad et al [21] have shown a simplified version of the RETAIN architecture that significantly predicted preterm birth and enabled individual-level prediction explanations at the visitation level and medical code level (*International Classification of Diseases, Ninth Revision* [*ICD-9*] or *ICD-10* codes). Rasmy et al [23] have adapted a language model that combined the RETAIN model with two independent EHR databases; this model achieved a high degree of accuracy in predicting both heart failure and the onset of pancreatic cancer.

In this study, we examined the performance of an interpretable deep learning model using longitudinal nursing narratives to predict prolonged LOS and extracted the significant nursing narrative features to better understand the prediction model.

## Methods

### Ethical Considerations

This study was approved by the Seoul National University Bundang Hospital (SNUBH) Institutional Review Board (B-2011/646-104 for model development; B-2103-675-101 for physician comparison).

### Setting

*ICD-10* diagnosis code C56 was used to identify the study population. Data were retrospectively collected from patients admitted to the SNUBH for first-time ovarian cancer surgery between 2014 and 2021.

We divided the data into two parts by period: the internal data set (collected between January 2014 and September 2020) and the external validation data set (collected between October 2020 and February 2021) [24]. We chose the most recent 5 months of data as the external validation data set. The internal data set was used for training, validating, and testing the model, while the external data set was used for evaluating the final performance of the model and comparing the results with the physician’s assessment.

The exclusion criteria included readmission, admission with postoperative LOS <3 days, and patients who underwent surgery <20 times in the internal data set (Figure S1 in Multimedia Appendix 1). Postoperative LOS was chosen as the outcome variable because in-hospital LOS can be affected by several nonoperational factors such as patient characteristics or social circumstances, type of admission, patient place of residence, emergencies, or weekend admissions [9,25,26]. The postoperative LOS was defined as the number of days from the date of the index operation to the date of discharge, where the date of the index operation, denoted as day 0, was defined as the date in any of the operation-related nursing narratives. For example, if a patient was discharged on day 0, the postoperative LOS was 1.

### Nursing Narratives

We extracted nursing narratives chronologically. At SNUBH, nursing narratives are easily integrated into a structured database because individual features are mapped to unique codes [11,27]. For instance, the nursing narrative “checked the vital signs” is mapped to code N1, whereas “no dizziness” is mapped to code N2. A nurse enters patient statuses in the EHR system by searching nursing narratives using keywords such as “vital” or “dizzy” and selecting the appropriate nursing narratives from the list of related narratives. Some nursing narratives allow for the inclusion of additional information such as body temperature or free text [14]. Consequently, patient information was entered as a combination of unique codes (eg, N1, checked the vital signs, or N2, checked whether the patient felt dizzy), code entry time, and a specific value such as body temperature. These structured nursing narrative sets allowed us to retrieve patient information without the need for natural language preprocessing.

### RETAIN Architecture

Prolonged LOS was defined as events ≥10 postoperative days, which was the third quantile of postoperative LOS in both the internal and external validation data sets. Our results showed that the volume of nursing narratives entered within 3 postoperative days was high and tended to decrease afterward; therefore, we decided to use patient information within 3 postoperative days, that is, from day 0 to day 2 (Figure S2 in Multimedia Appendix 1). The extracted time series of nursing narratives and corresponding unique codes were inverted to 3D arrays (patients, postoperative days, and nursing narratives’ unique codes).

The internal data set was randomly split, allocating 60% (n=192) of participants to the training set and 20% (n=64) of participants each to the validation and testing sets. The training set was used to train the models, the validation set was used to determine the values of the hyperparameters that increase the area under the receiver operating curve (AUC), and the test set was used to evaluate the performance of the best model. The best model was also applied to the external validation data set. Therefore, the performance of the best model was measured twice (the test set of the internal data set and the external validation data set). Furthermore, we compared the performance of the external validation data set with the physician’s assessment. The RETAIN model was constructed with two neural attentions that can identify influential nurse visits and meaningful features. The RETAIN model uses linear embedding to enhance interpretability. The contribution score was calculated using visit-level attention weights, variable-level attention weights, and embedding weights.

The default settings for RETAIN were used. L2 regularization for the final classifier weight, input embedding weight, and alpha-generating weight was set to 0.0001 for all models. Following a learning process using batch sizes of 8, 16, and 32, the model with the highest AUC performance on the test set was selected as the best model. If models had the same AUC value, the one with the highest sensitivity was selected as the best model.

### Model Interpretation and Influential Features Extraction

The RETAIN model reported the contribution scores that represented the extent to which each feature contributed to the prediction. In this study, features with a high contribution score were associated with a high likelihood of prolonged LOS. We identified the input features with high contribution scores as influential features, which showed a significant difference between prolonged and short LOS via a *t* test with a *P* value cutoff of .05.

### Comparison Between the Deep Learning Model and a Physician’s Expert Clinical Assessment

We compared the deep learning model and a physician’s assessment vis-à-vis their predictive capability for prolonged LOS. A gynecologic oncologist with 15 years of experience reviewed patients’ demographics, progress notes, surgical reports, and clinical notes available within 3 postoperative days. Blinded to the final discharge date, the physician predicted whether patients would experience prolonged LOS. The DeLong test was used to compare the AUCs of the deep learning model and a physician assessment [28].

### Visualization and Statistical Analysis

Statistical analyses were performed using Python (version 3.9.13; Python Software Foundation), RETAIN (version 1.0; Edward Choi) [16], and R (version 4.0.5; R Foundation for Statistical Computer) software. The influential features were visualized using the ComplexHeatmap package in R software.

## Results

### Patient Characteristics

This study retrospectively enrolled 354 patients (n=320 in the internal data set and n=34 in the external validation data set; mean age 54, SD 13 years; Table S1 and Figure S1 in Multimedia Appendix 1). A total of 51,603 nursing narratives in the internal data set composed the model inputs. Patients in the prolonged LOS group were older (for instance, in the internal data set, the mean age was 57, SD 12 years and 52, SD 14 years in the prolonged LOS and short LOS groups, respectively; *P*=.002) and had higher total nursing narrative volumes (mean 188, SD 62 vs mean 150, SD 33 narratives within 3 postoperative days; *P*<.001). The. nursing narrative entries per nurse visit were similar (mean 5.6, SD 8.4 vs mean 5.9, SD 7.9 for the prolonged LOS and short LOS groups, respectively; *P*=.64), but more frequent nurse visits were observed in the prolonged LOS group (mean 33, SD 9 vs mean 25, SD 9 visits within 3 postoperative days; *P*=.03).

### Prediction of Prolonged LOS via RETAIN

The experimental scheme is shown in Figures S2 and S3 in Multimedia Appendix 1. The RETAIN model was developed using the internal data set, while the model’s performance was calculated and compared to a physician’s expert clinical assessment using the external validation data set. The predictive contribution score derived from the RETAIN model indicates the probability of prolonged LOS. Patients with a final predictive score >0.5 were classified as expected prolonged LOS events. The RETAIN model reported the scores for each patient and nursing narrative; these scores were used to determine highly influential nursing narratives. We determined potent nursing narratives for each patient based on patient-wise normalization scores, after applying normalization using a patient-centric mean and SD. Thereafter, influential nursing narratives were defined as those consistently showing a statistically significant difference in the raw contribution score between the prolonged and short LOS groups.

Table 1 shows a comparison between the model’s performance and a physician’s expert clinical assessment, considering various nursing narrative sets. The model trained with nursing narratives showed an AUC of 0.81. The deep learning model performed better than the physician’s assessment (AUC 0.58; *P*=.02; Figure S4 in Multimedia Appendix 1).

**Table 1.** Model performance on the internal and external validation set.

Data set and model		AUC^a^	Accuracy	Sensitivity	Specificity	*F*_1_-score	*P* value^b^
**Internal data set**							
	RETAIN^c^ with nursing narratives	0.76	0.80	0.55	0.91	0.63	N/A^d^
**External validation data set** ^**e**^							.02
	RETAIN with nursing narratives	0.81	0.85	0.44	1.00	0.62	
	Physician assessment	0.58	0.65	0.44	0.72	0.40	

a AUC: area under the receiver operating curve.

b The DeLong test was conducted to compare the AUCs of the RETAIN model and physician assessment.

c RETAIN: Reverse Time Attention.

d N/A: not applicable.

e The RETAIN model performance and physician assessment were compared using the external validation data set. A total of 34 patients were available for 3 postoperative days.

### Influence of Nurse Visits on the Prediction of Prolonged LOS

**Figure 1.**
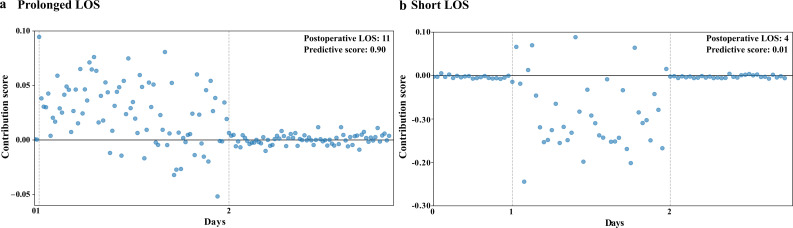
Highly influential nursing narrative (NN) examples presenting the differences between the prolonged and short LOS groups’ contribution score graphs. NNs are arranged in chronological order, while the corresponding scores are represented as dots. The predictive score indicates the probability of prolonged LOS, which was estimated by the Reverse Time Attention model, with (A) a postoperative LOS of 11 days (predictive score: 0.90) and (B) a postoperative LOS of 4 days (predictive score: 0.01). LOS: length of stay.

Examples of contribution score graphs were visualized for patients in the prolonged and short LOS groups (Figure 1). As expected, the patients in the prolonged LOS group exhibited high contribution scores. Nurse visits on the first postoperative day (ie, day 1) were identified as highly influential because nursing narratives entered on that day exhibited higher contribution scores.

**Figure 2.**
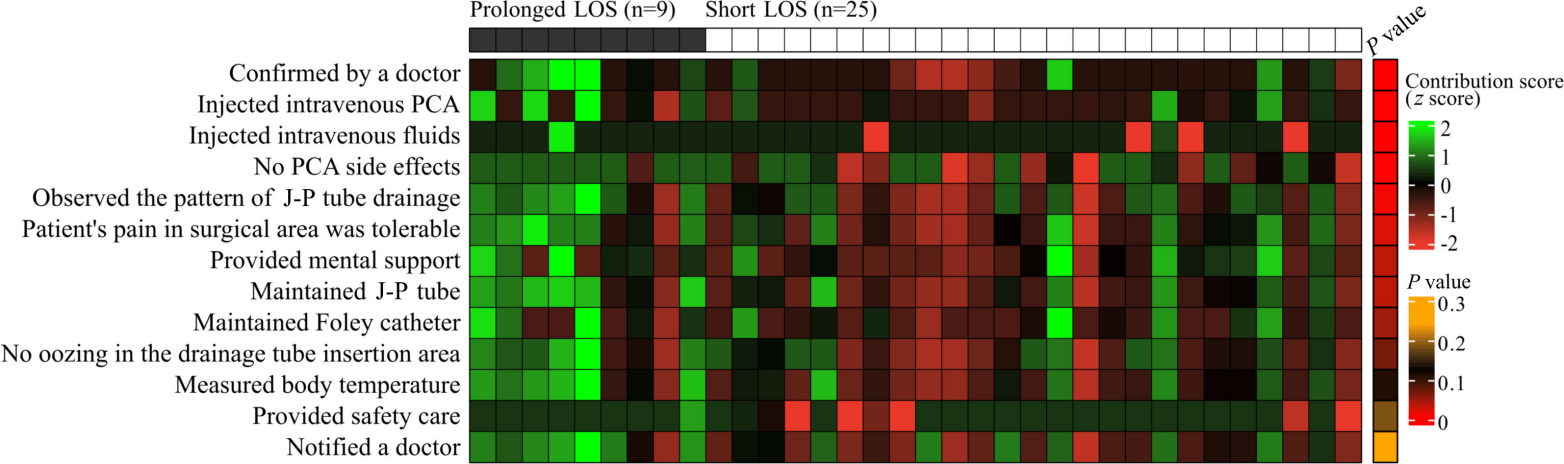
Heat map visualizing the contribution scores of highly influential nursing narratives (NNs). NN-level normalized contribution scores were calculated for patients of the external data set. The *P* value represents the results of the *t* test for raw contribution score comparison between the prolonged LOS and short LOS groups. J-P: Jackson-Pratt; LOS: length of stay; PCA: patient-controlled analgesia.

Highly influential narratives showing statistically significant differences in contribution scores between the prolonged and short LOS groups included the following: “confirmed by a doctor,” “injected intravenous patient-controlled analgesia [PCA],” “injected intravenous fluids,” “no PCA side effects,” “observed the pattern of Jackson-Pratt [J-P] tube drainage,” “patient’s pain in surgical area was tolerable,” “provided mental support,” “maintained J-P tube,” “maintained Foley catheter,” “no oozing in the drainage tube insertion area,” “measured body temperature,” “provided safety care,” and “notified a doctor” (Figures2  3). The three most influential narratives (according to their lower *P* values) were “confirmed by a doctor,” “injected intravenous PCA,” and “injected intravenous fluids” (Table 2), whose contribution scores were visualized by *t*-distributed stochastic neighbor embedding (Figure 4).

**Figure 3.**
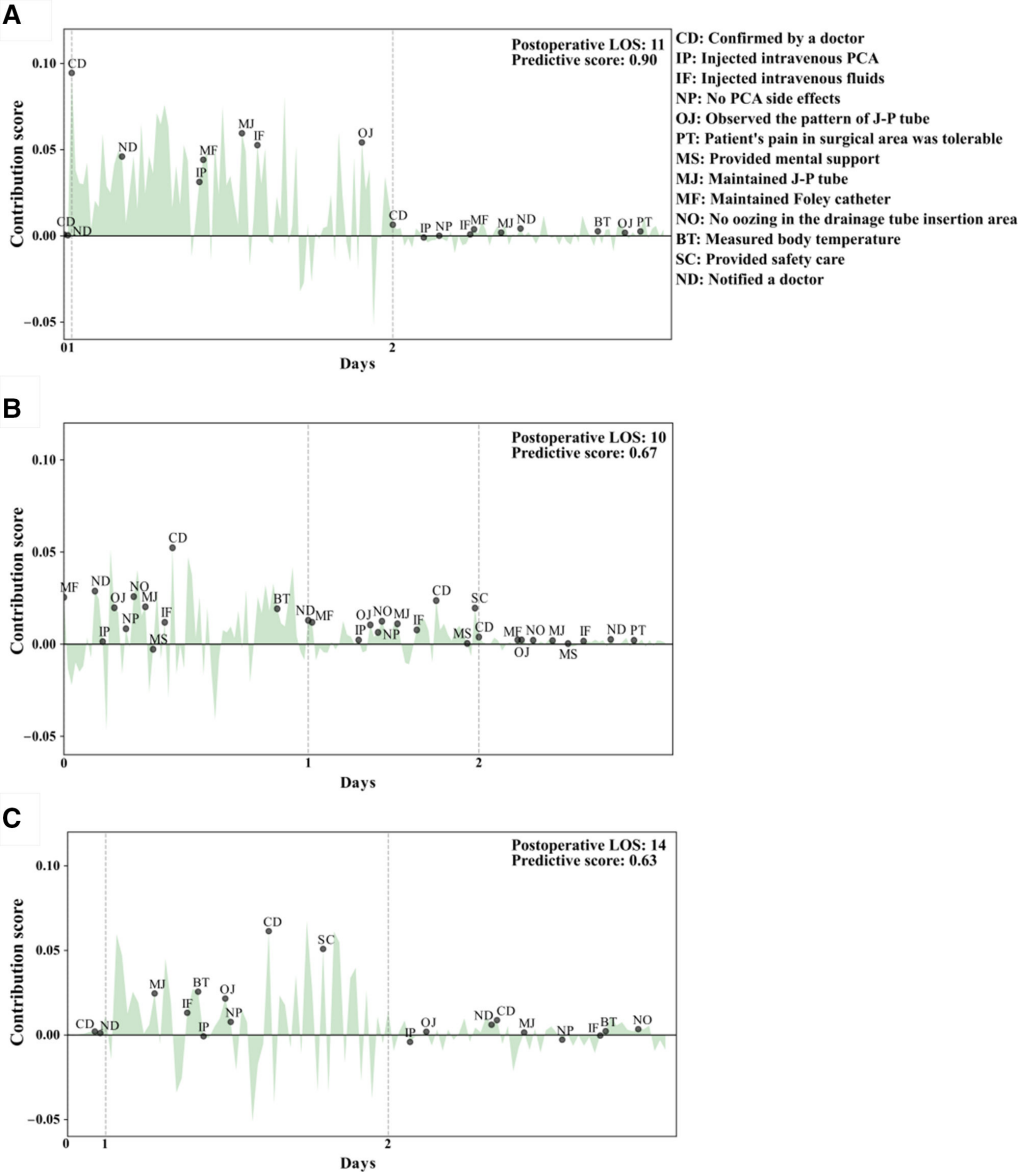
The contribution score graph highlights highly influential nursing narratives (NNs) of the prolonged LOS group, with the NNs arranged in chronological order. The areas of the corresponding contribution scores are filled. The predictive score indicates the probability of a prolonged LOS. The patients with predictive scores >0.5 were classified as expected prolonged LOS. The most influential NNs are represented as orange dots. (A) Postoperative LOS: 11 days; predictive score: 0.9; (B) postoperative LOS: 10 days, predictive score: 0.67; (C) postoperative LOS: 14 days, predictive score: 0.63. J-P: Jackson-Pratt; LOS: length of stay; PCA: patient-controlled analgesia.

**Table 2.** Contribution scores of influential nursing narratives.

Nursing narratives	Internal data set (n=320)			External validation data set (n=34)		
	Prolonged LOS^a^, mean (SD)	Short LOS, mean (SD)	*P* value^b^	Prolonged LOS, mean (SD)	Short LOS, mean (SD)	*P* value^b^
Confirmed by a doctor	0.044 (0.038)	–0.001 (0.021)	<.001	0.030 (0.033)	0.000 (0.020)	.002
Injected intravenous PCA^c^	0.012 (0.046)	–0.074 (0.041)	<.001	–0.030 (0.047)	–0.076 (0.035)	.003
Injected intravenous fluids	0.012 (0.044)	–0.066 (0.036)	<.001	–0.009 (0.043)	–0.057 (0.036)	.003
No PCA side effects	–0.002 (0.058)	–0.092 (0.052)	<.001	–0.037 (0.055)	–0.094 (0.044)	.004
Observed the pattern of J-P^d^ tube drainage	0.016 (0.039)	–0.039 (0.033)	<.001	–0.001 (0.038)	–0.039 (0.034)	.007
Patient’s pain in the surgical area was tolerable	0.019 (0.036)	–0.031 (0.027)	<.001	–0.011 (0.025)	–0.034 (0.024)	.02
Provided mental support	–0.004 (0.043)	–0.065 (0.060)	<.001	–0.009 (0.026)	–0.053 (0.056)	.03
Maintained J-P tube	0.019 (0.036)	–0.035 (0.031)	<.001	–0.005 (0.040)	–0.034 (0.031)	.03
Maintained Foley catheter	0.019 (0.019)	0.002 (0.007)	<.001	0.011 (0.017)	0.003 (0.006)	.045
No oozing in the drainage tube insertion area	0.011 (0.022)	–0.008 (0.017)	<.001	0.001 (0.004)	–0.009 (0.016)	.06
Measured body temperature	0.008 (0.025)	–0.018 (0.027)	<.001	0.003 (0.009)	–0.007 (0.017)	.11
Provided safety care	0.026 (0.027)	0.006 (0.012)	<.001	0.020 (0.019)	0.011 (0.016)	.18
Notified a doctor	0.021 (0.019)	0.004 (0.010)	<.001	0.012 (0.017)	0.006 (0.010)	.24

a LOS: length of stay.

b *P* values represent the results of the *t* test for raw contribution scores compared between the prolonged and short LOS groups.

c PCA: patient-controlled analgesia.

d J-P: Jackson-Pratt.

**Figure 4.**
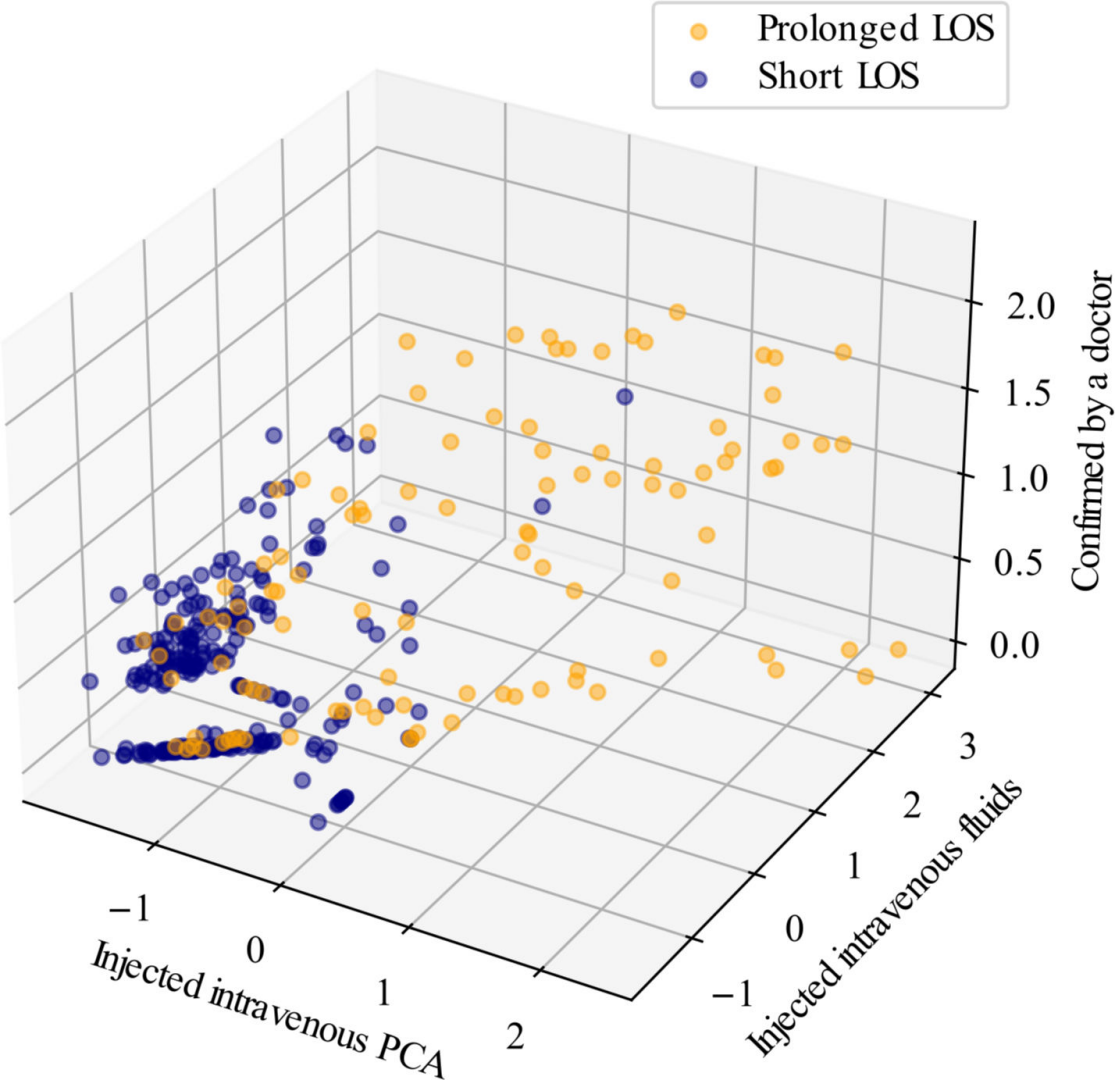
T-distributed stochastic neighbor embedding plot using the top three highly influential nursing narratives; the contribution score was estimated from the external data set. LOS: length of stay; PCA: patient-controlled analgesia.

Once the three most influential nursing narratives were identified, we further investigated the total number of entries and the first entry time since the handoff. The “confirmed by a doctor” narrative reoccurred in the prolonged LOS group (mean 5.8, SD 4.4 vs mean 3.1, SD 2.3 nursing narratives in the prolonged and short LOS groups, respectively) and was entered earlier (Table S2 in Multimedia Appendix 1). Conversely, the narratives “injected intravenous PCA” and “injected intravenous fluids” exhibited similar entry values but were entered a few hours later in the prolonged LOS group.

## Discussion

### Principal Results

In this study, a RETAIN model was used to predict postoperative LOS using nursing narratives. The model achieved a higher AUC value of 0.81 compared to the physician assessment’s AUC of 0.58 (*P*=.02). Highly influential nursing narratives were identified that differed in their contribution scores between the prolonged and short LOS groups, including confirming by a doctor, administering intravenous PCA, and providing intravenous fluids.

To our knowledge, this is the first study to extract nursing narratives’ influential features by normalizing contribution scores estimated via RETAIN. By investigating these influential features, we discovered that the volume and timing of individual narratives are key factors. The likelihood of prolonged LOS increases if a physician must check the patient more often or if intravenous fluids or intravenous PCA are administered late. This degree of interpretability was not achievable in previous studies that relied on volume-centric statistical methods and conventional deep learning models.

### Strengths

This study demonstrated that nursing narratives can accurately predict the postoperative LOS of patients who underwent surgery for ovarian cancer. We implemented an interpretable deep learning model to identify highly influential nursing narratives. Notably, nursing narratives entered one day after surgery were the primary predictors for prolonged LOS.

Nursing narratives, serving as proxies for the care given to patients, demonstrated predictive value for LOS. Nursing narratives thus reflect the actions and interventions carried out by health care professionals. By identifying highly influential nursing narratives and presenting the different action timing and volume of each narrative, we enhanced the model’s interpretability and showed that the relevant nursing activities could serve as indicators for LOS.

These findings support other studies that have shown that nursing notes may predict short-term patient outcomes more accurately than physician notes [29,30]. Nurses frequently summarize patients’ situations by describing their symptoms, as well as their nursing actions and responses, without the restriction of structured forms [31-33]. Thus, nursing notes serve as a snapshot of patients’ current statuses and exhibit a higher degree of freedom compared to physicians’ notes, which provide a problem-focused summary. In a prospective cohort of patients who are critically ill, nurses predicted in-hospital mortality slightly more accurately than physicians, whereas the latter predicted long-term outcomes more accurately [29]. Huang et al [30] applied natural language processing to free-text nursing notes to predict multiple outcomes, including prolonged hospital stay or mortality, using the Multiparameter Intelligent Monitoring of Intensive Care III. This study also acknowledged the superior predictability value of nursing notes over physicians’ notes when using refined features within the first 48 hours of admission. However, none of these studies presented the additional interpretation of specific nursing notes.

Furthermore, this study showed that the total volume of nursing narratives is a significant factor for prolonged LOS, which was consistent with previous studies conducted in different settings. Schnock et al [34] have conducted a multicenter qualitative study in intensive and acute care units to discover nursing documentation patterns indicating recovery patterns. Woo et al [35] have used the natural language processing of nursing notes from patients admitted to home care and found that the frequency of wound infection–related text in nursing notes increased before hospitalization or emergency department visits. However, these studies faced a common barrier to using nursing notes: the extraction of standardized information. Accordingly, there is a significant need for health care providers to standardize nursing assessments and free-text notes [30].

We showed that the nursing narratives “confirmed by a doctor,” “injected intravenous PCA,” and “injected intravenous fluids” were relevant to a prolonged stay for patients with surgical procedures. These narratives suggested that a patient’s condition is complicated, and additional support for pain management or fluid management was required. Timely communication and collaboration between nursing and medical staff, effective pain management, and appropriate fluid management are important considerations in surgical patient care, which can impact the LOS and overall patient outcomes.

### Limitations

This study had several limitations. First, this study was based on data from a single-hospital EHR system. The EHR system at SNUBH allows for the standardization of nursing narratives, which enables the creation of a structured database. However, in most hospitals, free-text nursing notes are common; therefore, the preprocessing of natural language is required to generalize this study’s findings. As a starting point, it is worthwhile to examine the highly influential nursing narratives identified in our study. Second, we chose nursing narratives entered within a 3-day postoperative interval, which can be shortened in future studies. For example, 2-day postoperative data have been used in several studies [9,30]. Furthermore, a strategic patient care plan that combines a short-interval model and a long-interval model could be developed. Third, this study’s sample size was small, while the model was developed in a single-disease setting. To consider the dependency of nursing narratives according to different surgery and patient settings, transfer learning (in which a model trained in a larger population is fine-tuned with an independent surgery setting) can be considered. Future studies with multiple hospital settings and multimodal features are required [36]. Fourth, like other machine learning models, training and testing a model requires a large amount of data, and multiple validation sets are needed to avoid overfitting [37]. In addition, as the RETAIN model receives input values for each variable and visit level, it may be difficult to apply the model to unstructured data such as free text or data that cannot be classified by date. Finally, it is important to acknowledge that physician assessments were done retrospectively, potentially not capturing dynamic clinical situations.

### Future Perspectives

Collecting a larger data set that includes a wider range of patients and additional predictors such as laboratory data or comorbidity information is essential. We firmly believe that integrating nursing narratives with broader information, including physician assessments, can lead to a better prediction model.

### Conclusions

In this study, an interpretable prediction model for a longer postoperative LOS was developed using nursing narratives. The day after surgery was the most critical time for prediction, and influential nursing narratives were revealed. Although nursing narratives serve as proxies for the care given to patients, our study suggests that they have the potential to be predictors for LOS. The developed model can help identify patients with a prolonged hospital stay at the right time, thereby improving patient care and reducing hospital management burden. To strengthen the evidence supporting the predictive value of nursing narratives, either alone or in combination with broader information such as physician assessment, a larger data set would be beneficial.

Our study highlights that nursing narratives are predictors for prolonged LOS in patients undergoing ovarian cancer surgery. We emphasize the comprehensive nature of nursing actions and their timing in predicting patient outcomes and suggest methods to incorporate into a prediction model.

## Supplementary material

10.2196/45377 Multimedia Appendix 1 Supplementary material.

## Abbreviations

AUC: area under the receiver operating curve
EHR: electronic health record
*ICD-9*: *International Classification of Diseases, Ninth Revision*
J-P: Jackson-Pratt
LOS: length of stay
PCA: patient-controlled analgesia
RETAIN: Reverse Time Attention
SNUBH: Seoul National University Bundang Hospital
