# Supplementary material for: Predicting Postoperative Hospital Stays Using Nursing Narratives and the Reverse Time Attention (RETAIN) Model: Retrospective Cohort Study
Source: JMIR Med Inform. 2023 Dec 19;11:e45377. doi: 10.2196/45377 (PMC10763991; doi:10.2196/45377)
Supplement: Multimedia Appendix 1 [file medinform-v11-e45377-s001.docx]

**Supplementary Material**

**
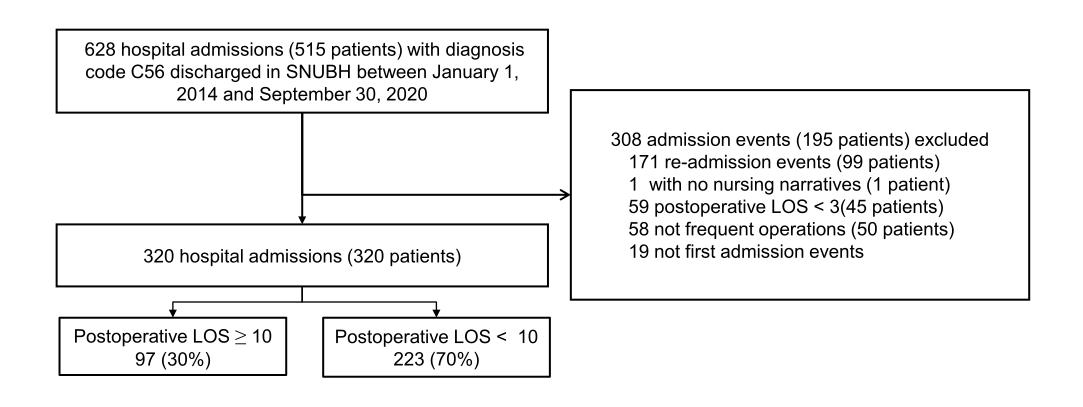
**

Figure S1. Flow diagram of the internal dataset

*Note.* LOS = length of stay; SNUBH = Seoul National University Bundang Hospital.

**
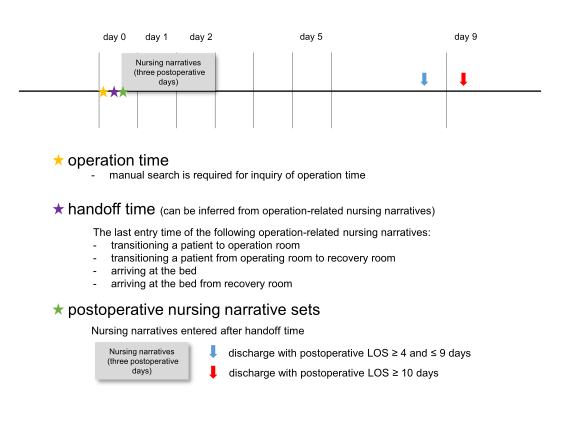
**

Figure S2. Definition of nursing narrative sets

Identifying the end of the operation time usually requires a manual search of the operation note. Instead, we chose operation-related nursing narratives as follows: transitioning a patient to the operating room, transitioning a patient from the operating room to the recovery room, arriving at the bed, and arriving at the bed from the recovery room. Handoff time was defined as the last entry time of operation-related nursing narratives.

**
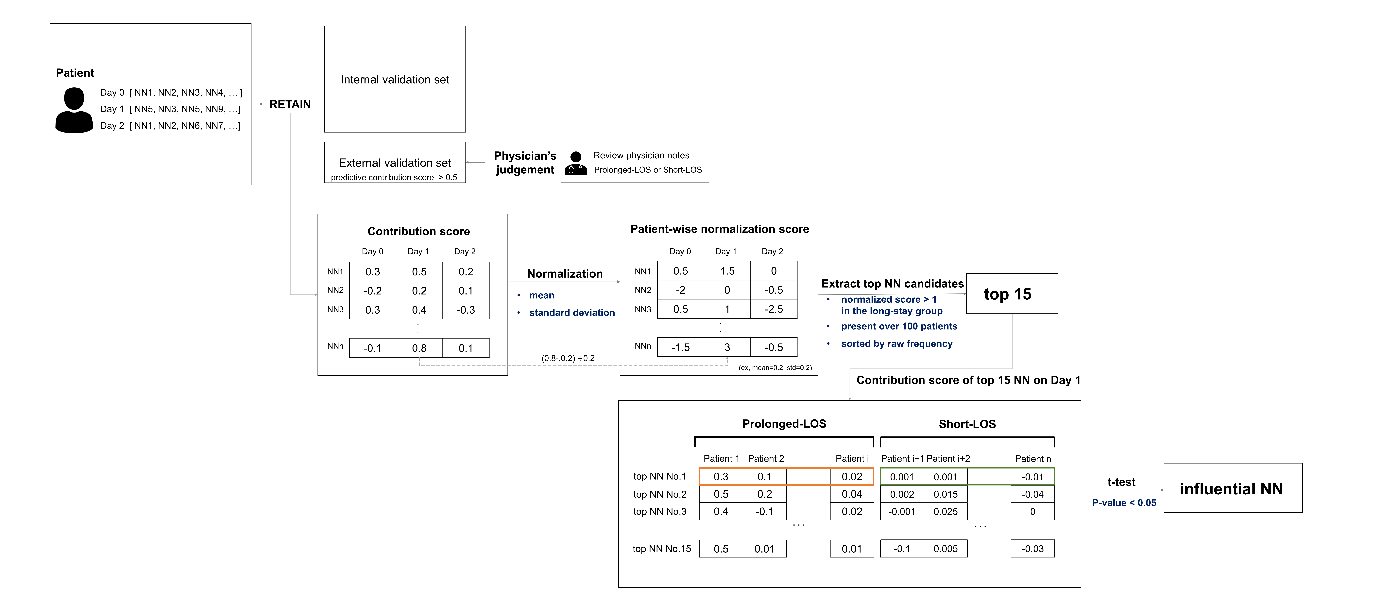
**

Figure S3. Overview of experiments using the RETAIN model and analysis plan

*Note.* LOS = length of stay; NN = nursing narratives; RETAIN = REverse Time AttentIoN model.


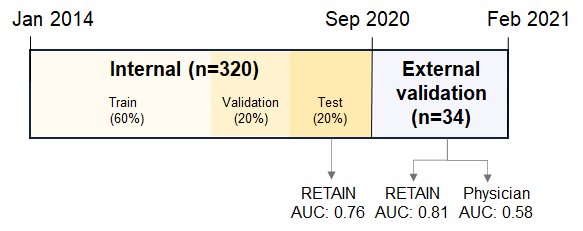


Figure S4. The timeline of internal and external validation data and model performance

Table S1 Patient characteristics

|  | Internal dataset  (n = 230) | | | | External dataset  (n = 34) | | |
| --- | --- | --- | --- | --- | --- | --- | --- |
|  | Prolonged-LOS  (n = 97) | Short-LOS  (n = 223) | | P-value^1^ | Prolonged-LOS  (n = 9) | Short-LOS  (n = 25) | P-value^a^ |
| **Length of stay (days)** | 19 (12, 21) | 8 (6, 9) | | <.001 | 13  (12, 14) | 7  (6, 8) | <.001 |
| **Postoperative length of stay (days)** | 15 (11, 17) | 6 (6, 7) | | <.001 | 12  (11, 13) | 6  (5, 7) | <.001 |
| **Age (years)** | 57 (50, 66) | 52 (45, 60) | | .002 | 70  (65, 76) | 49  (43, 56) | <.001 |
| **Total no. of nursing narratives^b^** | 188 (143, 216) | 150 (128, 171) | | <.001 | 188 (155, 208) | 148 (119, 167) | .008 |
| **Total no. of nurse visits^2^** | 33 (26, 41) | 25 (22, 29) | | .028 | 34 (25, 41) | 26 (20, 30) | <.001 |
| **No. of nursing narratives per nurse visit** | 5.6 (1, 4) | 6.0 (1, 6) | .635 | | 5.5 (1, 5) | 5.8 (1, 7) | .036 |

*Note.* Data are mean (interquartile range) values

^a^t-test was conducted.

^b^Total number of nursing narratives and nurse visits were within three postoperative days, that is, day 0 to day 2.

Table S2 Highly influential nursing narratives characteristics

|  | | Internal dataset (n = 230) | | | External dataset (n = 34) | | |
| --- | --- | --- | --- | --- | --- | --- | --- |
|  | | Prolonged-LOS (n = 97) | Short-LOS (n = 223) | P-value^1^ | Prolonged-LOS (n = 9) | Short-LOS (n = 25) | P-value^a^ |
| **Total number of nursing narratives^b^** | | | | | | | |
|  | Confirmed by a doctor | 5.82 (3, 8) | 3.11 (2,4) | <.001 | 6.25 (2, 8) | 2.68 (1, 3) | .003 |
|  | Injected intravenous PCA | 4.83 (4, 6) | 4.76 (4, 6) | .078 | 5.00 (4, 6) | 4.33 (4, 5) | .211 |
|  | Injected intravenous fluid | 4.60 (4, 6) | 4.22 (3, 5) | .754 | 4.00 (3, 5) | 3.64 (3, 4) | .535 |
| **First entry time from handoff time (hours)** | | | | | | | |
|  | Confirmed by a doctor | 7.68 (0.00, 12.00) | 14.60 (2.00, 23.00) | <.001 | 9.88 (1.00, 11.75) | 19.45 (4.00, 29.75) | .166 |
|  | Injected intravenous PCA | 8.46 (3.50, 11.00) | 6.67 (3.00, 8.00) | .038 | 9.89 (5.00, 14.00) | 8.33 (3.75, 11.25) | .533 |
|  | Injected intravenous fluid | 10.18 (4.00, 13.00) | 7.97 (3.00, 11.00) | .029 | 12.00 (5.00, 15.00) | 10.04 (4.00, 12.00) | .616 |

*Note.* LOS = length of stay; PCA = patient-controlled analgesia.

Data are presented as means (interquartile range).

^a^t-test was conducted.

^b^Data were obtained from three postoperative days, that is, day 0 to day 2.

Table S3 Abbreviation in Figure 3

| Nursing narrative | Abbreviation |
| --- | --- |
| **Confirmed by a doctor** | CD |
| **Injected intravenous PCA** | IP |
| **Injected intravenous fluids** | IF |
| **No PCA side effects** | NP |
| **Observed the pattern of J-P drainage** | OJ |
| **Patient's pain in surgical area was tolerable** | PT |
| **Provided mental support** | MS |
| **Maintained J-P tube** | MJ |
| **Maintained Foley catheter** | MF |
| **No oozing in the drainage tube insertion area** | NO |
| **Measured body temperature** | BT |
| **Provided safety care** | SC |
| **Notified a doctor** | ND |

*Note.* PCA = patient-controlled analgesia; J-P = Jackson-Pratt
